# Supplementary figures and images for: Evaluation of Anti-Biofilm Activity of Mouthrinses Containing Tannic Acid or Chitosan on Dentin In Situ
Source: Molecules. 2021 Mar 3;26(5):1351. doi: 10.3390/molecules26051351 (PMC7961503; doi:10.3390/molecules26051351)

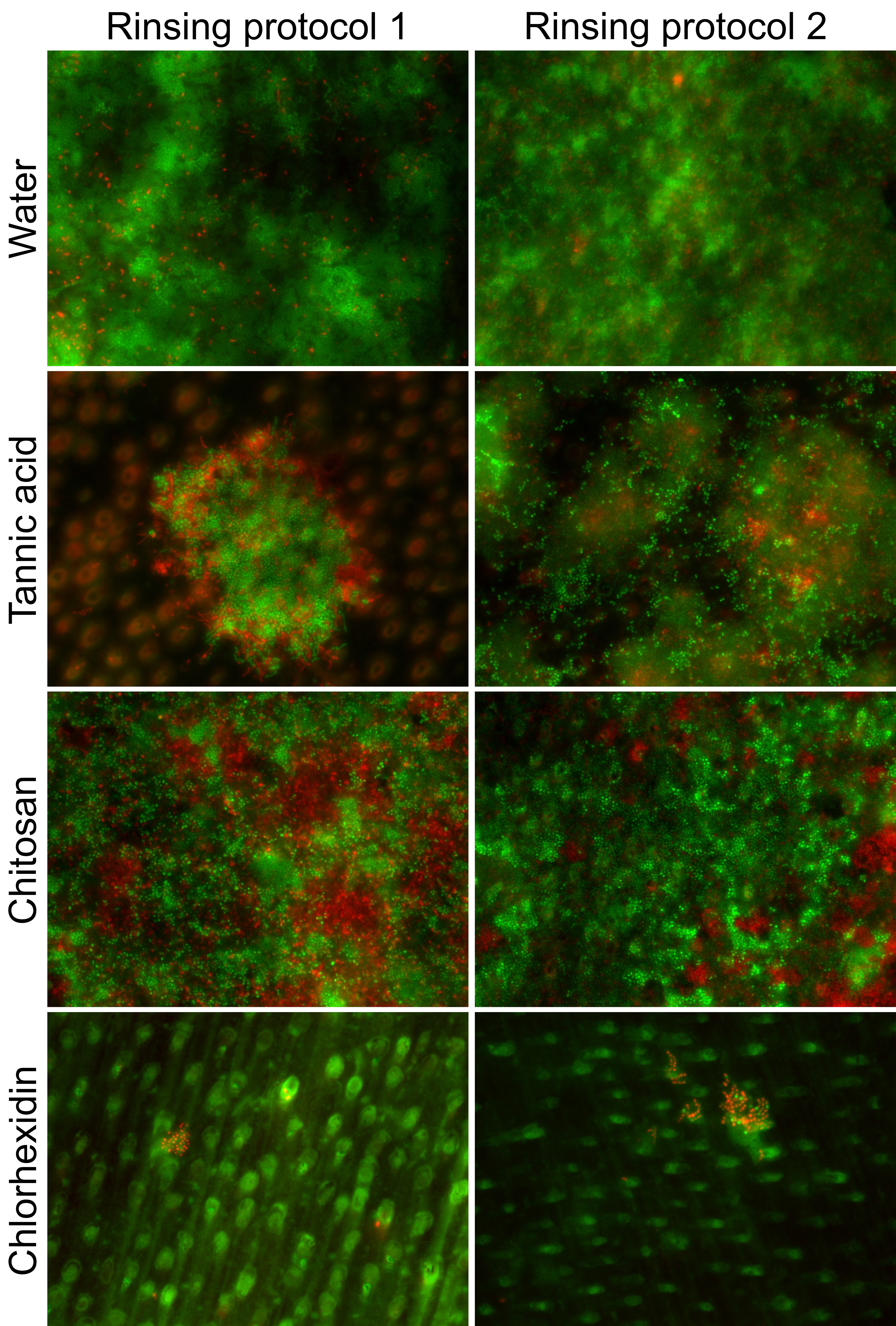

Supplement: Supplementary file 1 [file molecules-26-01351-s001.zip › figure s1.jpg]
